# Work-related Hazards Among Pathologists and Residents of Pathology:Results of a Cross-sectional Study in Iran

**DOI:** 10.30699/IJP.2021.132380.2473

**Published:** 2021-05-09

**Authors:** Maryam Kadivar, Elaheh Kabir-Mokamelkhah, Zohreh Habibi-Shams

**Affiliations:** 1 *Department of Pathology, Faculty of Medicine, Iran University of Medical Sciences, Tehran, Iran*; 2 *Occupational Medicine Research Center (OMRC), Iran University of Medical Sciences, Tehran, Iran*

**Keywords:** Occupational health, Pathologists, Workplace

## Abstract

**Background & Objective:**

Pathologists as medical professionals involved in the diagnosis and planning of therapies in many diseases are exposed to occupational hazards in workplaces. Hence, we aimed to determine the occupational health problems among Iranian pathologists in this cross-sectional study.

**Methods:**

This cross-sectional study was conducted among the Iranian pathologists. The data required for this study was collected through a self-reported questionnaire containing 48 questions about major occupational health problems, including musculoskeletal problems, visual disorders, workplace characteristics, health behavior, and other medical conditions.

**Results:**

Among the study participants (N=350), 87.4% presented with musculoskeletal disorders in the past year, with the neck as the most common location of pain (71%). Musculoskeletal pain was significantly higher in those working with the computer for more than 5 hours per day (*P*=0.007). Furthermore, 273 (78%) participants reported visual refractive errors, and myopia was the most common error (53%). Acute injuries were reported in 263 (75%) participants, and the cutting injury had the highest frequency (56.6%). Depression was reported in 54 (15.4%) of the participants, followed by burnout (10.3%) and hypertension (4%). Intolerance reactions to formalin were reported by 222 (63.6%) and were significantly more frequent among the residents (*P*<0.001). The residents were more prone to musculoskeletal pain (*P*=0.002) and injury (*P*=0.026).

**Conclusion:**

We observed a noticeable prevalence of health risks, including musculoskeletal problems, visual disturbances, injuries, and ergonomic problems among the Iranian pathologists. Solving these problems demands thorough prevention and personal protection, as well as educational programs with more attention toward optimization of ergonomics in the workplace and awareness about chemical and biological hazards.

## Introduction

Pathology is one of the oldest and major fields of medicine that deals with the diagnosis and prognosis of malignancies (1). In the routine work of pathologists, exposure to contaminants, germs, chemicals, and injury is frequent, and it can even result in their death (2, 3). The literature is full of case reports of various accidents, infections, or exposures that happened to the pathologists, and not all of them ended in a good fate (4-6). Many other problems also develop because of the working environment and posture, such as long working hours with a microscope in a rigid sitting position (7-9). Musculoskeletal disorders (MSD) are prevalent among medical laboratory professionals, and neck and back are the most common affected sites (10). Most of these disorders result from awkward posture during work (7, 11). Long hours of working with the microscope and computer, poor ergonomics, and not using standard equipment at workplaces predispose the pathologists to musculoskeletal problems (7, 8, 12, 13). Unfortunately, there is a lack of awareness and assessment of these issues at the workplace, and this puts the pathologists in a higher risk of developing occupational problems (7). Exposure to chemicals, particularly formaldehyde, can cause various comp-lications, from allergy and irritative reactions to malignancies (14-16). On the other hand, current evidence shows that pathologists may also be more susceptible to developing the brain, hematopoietic, and lymphatic malignancies that occur due to exposure to chemicals, mostly formaldehyde (2-18). Therefore, occupational health of pathologists and determining its related issues is a crucial topic (11).

Few studies have discussed the occupational health risks in pathologists. In a study conducted by Evan George, practicing pathologists were identified to be at high risk for the development of MSDs of the neck, upper back, lower back, shoulders, and upper extremities related to cumulative trauma (7). 

The study of Fritzsche *et al.* is a landmark study in this regard that evaluated the occupational health risks of 163 pathologists in Switzerland (19). Moreover, a review study conducted in 2014 on the prevalence of work-related musculoskeletal disorders among medical laboratory professionals showed that the overall prevalence was 40-60% with neck pain being more prevalent (18-78%) (20).

 In a study conducted by Jalali *et al.* In Iran regarding the chemical hazards in the workplace of pathologists, it was found that the physicians were exposed to very high levels of formaldehyde in their workplaces, which can cause respiratory failure (21). A systematic review study on pathologists in 2020 found that job satisfaction, stress, and burnout were associated with various occupational features, including elements of demand, support, and reward in these physicians (22).

 As mentioned, in most studies performed on pathologists, only one occupational risk factor has been investigated, and few studies have investigated all occupational risk factors. The results of a study conducted in 2020 in France showed that 38% of pathologists had musculoskeletal disorders in the last 6 months and 73.4% had visual disturbances, 33.3% of pathologists had been injured or had mucosal projections during macroscopic autopsy of specimens, and psychological disorders such as depression or burnout were reported by 16.7% of respondents (23); though this study is from a wealthy and developed country, and hence its results cannot be generalized to other countries. Additionally, data from the Middle East and particularly Iran on this topic is limited. Considering that most studies have been conducted in developed countries and in most studies only one occupational risk factor has been examined and not several risk factors have been studied together, we decided to determine the occupational health problems among the Iranian pathologists in a cross-sectional study.

## Material and Methods

In this cross-sectional study, all pathologists and residents of pathology who worked in a laboratory at the time of the study were invited to participate. The invitations were issued by referring to the Pathology Laboratories in Tehran or in-person invitation to the participants in the Annual Pathology Congress of Iran. The exclusion criterion was working in a non-laboratory setting at the time of the study. All the participants gave their consent to the use of data for research purposes. As a reward for participation, the participants were given a chance of taking part in a lottery for a reward at the end of the congress. The study protocol was reviewed and approved by the Research Board and the Committee of Medical Ethics at Iran University of Medical Sciences (IR.IUMS.FMD.REC.13797.211). 

The study instrument was a written 48-item questionnaire about occupational health, occupational hazards, characteristics of the working environment, and confrontation attitudes for occupational hazards. Occupational hazards included musculoskeletal disorders, visual disturbances, cutting injuries, psychological disorders, and adverse effects related to the long-term exposure to chemicals leading to hypersensitivity reactions or malignancy. This questionnaire was based on the study of Fritzsche *et al.* (24). The main questionnaire was translated into Persian by an experienced person and was given to several pathologists. Then, those questionnaires were re-evaluated and used as the validity and reliability were confirmed. Then collected data were transferred to an SPSS file and analyzed using SPSS version 21.0 (IBM Corp. released 2012. IBM SPSS Statistics for Windows, version 21.0. Armonk, NY: IBM Corp.). Categorical data were described as frequency (percentage) and compared between the groups using the Chi-square test. The quantitative variables were expressed as mean ± standard deviation and compared by student’s t-test or Mann–Whitney U test where applicable. Kolmogorov-Smirnov test was used to determine the normality of the data distribution. A P-value<0.05 was considered as statistically significant.

## Results

**Participants**

In this study, 350 individuals consisting of 267 pathologists and 83 residents of Pathology participated. Two hundred and thirty-seven participants were men (67.7%), and 155 (44.2%) were younger than 45 years. Almost 70% of the participants (n=245) worked more than 50 hours per week, and 150 individuals (42.8%) had a part-time job. Working with a microscope and computer were reported in 334 (95.4%) and 313 (89.4%) of the participants, respectively. The general characteristics of the participants are reported in Table 1. 

**Musculoskeletal Problems**

Our data showed that 306 participants (87.4%) experienced musculoskeletal pain, and the neck was the most commonly reported location of pain (71%; Table 2). Ninety-six participants had at least one day of absenteeism due to musculoskeletal pain. Overall, musculoskeletal pain was reported more significantly in the residents (*P*=0.002), and they significantly performed fewer stretching exercises or got restless than the pathologists get (*P*=0.007). Moreover, neck pain did not differ between the residents and specialists while back and lower limb pain were more reported by the specialists. Musculoskeletal pain was significantly higher in those who worked with the computer for more than 5 hours per day (*P*=0.007). However, only 115 participants (32.9%) had information about ergonomics and posture, and the use of ergonomic furniture or receiving information on ergonomics was not associated with the frequency of pain (*P*=0.163 and *P*=0.700, respectively). Younger age groups significantly reported higher rates of musculoskeletal disorders, as did the older ones (*P*=0.004). 

**Table 1:** The General characteristics of the participants

Parameter	Value (n=350)
Age group	
25-35 years	130 (37.1)
36-45 years	125 (35.7)
46-55 years	68 (19.4)
>55 years	27 (7.7)
Male gender, n (%)	237 (67.7)
Occupation	
Pathologist	267 (76.3)
Resident of pathology	83 (23.7)
Type of job	
Private	141 (40.3)
Public, academic	151 (43.1)
Public, non-academic	58 (16.6)
Working hours >50 hours per week	185 (52.8)
Full-time job	41 (11.7)
Daily use of microscope	334 (95.4)
Daily use of microscope >5 hours	149 (42.6)
Daily use of computer	313 (89.4)
Daily use of computer>5 hours	76 (21.7)
Ability to time management	253 (72.3)
Organized tasks	236 (67.4)
Prior knowledge on ergonomics	115 (23)

**Fig. 1:**
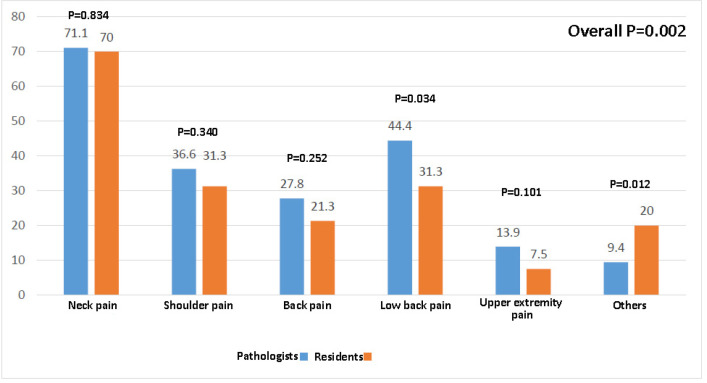
Comparison of musculoskeletal pain experience among the pathologists and residents

Working hours per week and duration of microscope use were also not associated with musculoskeletal problems (*P*=0.856 and *P*=0.205, respectively). Performing regular exercise was not related to musculoskeletal problems (*P*=0.385) (Figure 1). 

**Visual Errors**

 A total of 273 (78%) participants reported visual refractive errors, and myopia was the most prevalent form of error (53%; Table 2). However, 175 (50%) individuals had a visual error before their professional work. Refractive errors were more prevalent in women (*P*=0.024). Eye fatigue was significantly associated with using a microscope for more than 5 hours (*P*=0.023) and was more frequent in the residents (*P*=0.039). However, refractive errors were not associated with the duration of microscope use per day (*P*=0.077). 

**Injury**

An overall history of acute injuries during work was reported in 263 (75%) participants, and the cutting injury was the most common type (56.6%; Table 2). Splash into mucous membranes was the second most common type of injury. Neither the cutting injury, nor splash into mucous membranes, needle stick, and the overall injuries were associated with sex and type of job. Acute injuries (cutting) were also significantly more prevalent among the residents (*P*=0.026). Furthermore, the occurrence of these injuries within the past year was more prevalent among the residents (*P*<0.001). However, only 161 (46%) of the participants reported this event in the past year, and 18 (5.1%) reported a permanent injury. Intolerance reactions to formalin were reported by 222 (63.6%) participants, who were significantly more frequent among the residents (*P*<0.001). None of the participants were familiar with cut-resistant gloves. 

Reaction to formaldehyde was more significantly observed among the residents (*P*<0.001), but it did not differ between the sex groups (*P*=0.112). 

**Other Health Problems**

As regards other medical conditions, depression was reported in 54 (15.4%) of the participants, followed by burnout (10.3%) and hypertension (4%). Depression and burnout were significantly associated with each other (*P*<0.001), and 31 pathologists were affected by both (8%). Depression was significantly less frequent among pathologists who worked in university hospitals (*P*=0.034). Experience of burnout by participants who worked less than 50 hours per week and worked in a good atmosphere was significantly lower than that by the others (*P*=0.038 and *P*=0.014, respectively).

Five individuals had a history of malignancy; one case of lymphoma, one breast cancer, one colon cancer, and two individuals did not mention the site of malignancy. Hepatitis B vaccination was done in 339 (96.9%) of the participants, and 286 (81.7%) had received the Bacillus Calmette–Guérin (BCG) vaccine. Only one individual reported a positive purified protein derivative (PPD) skin test and latent tuberculosis. These data are summarized in Table 2. 

**Table 2:** The health status parameters in the studied pathologists

Parameter	Total (n=350)
*Musculoskeletal disorders*	
Musculoskeletal pain	306 (87)
Neck pain	217 (71)
Shoulder pain	106 (35)
Back pain	79 (26)
Lower back pain	124 (40)
Upper extremity pain	37 (12)
Pain in other organs	37 (12)
Absenteeism due to pain	96 (27)
Pain within the past month	221 (63)
Rest and exercise	152 (43)
Receiving ergonomic information	115 (32)
*Visual problems*	
Visual refractory errors	273 (78.0)
Myopia	185 (53.0)
Hyperopia	43 (12.3)
Other problems	45 (12.9)
Previous refractory error	175 (50.0)
Exacerbation of refractory error	180 (51)
Eye fatigue	214 (61.1)
*Injuries and exposures*	
Acute injury during work	263 (75.0)
Needle stick	62 (17.7)
Cutting injury	198 (56.6)
Splash into mucosal membranes	91 (26.0)
Injury within the past year	161 (46.0)
Permanent sequel	18 (5.1)
Reaction to formaldehyde	222 (63.3)
Known allergic reaction	142 (41)
History of allergy	296 (68.0)
*Other medical problems*	
Tuberculosis	1 (0.3)
Positive PPD skin test	1 (0.3)
Depression	54 (15.4)
Burnout	36 (10.3)
Hypertension	14 (4.0)
Diabetes mellitus	4 (1.1)
Malignancy	5 (1.4)
No health problem	235 (67.1)
HBV immunization	339 (96.9)
BCG vaccination	286 (81.7)

BCG: Bacillus Calmette–Guérin; HBV: Hepatitis B virus; PPD: Purified protein derivative

Over half of the pathologists (52.9%) reported working an average of more than 50 hours per week. About 90% of those pathologists with ≤50 hours work per week were employed on a part-time basis. Additionally, 95% of participating pathologists used a microscope on a daily basis, and 89% worked daily on a computer. Forty-two percent and 21% of pathologists reported working more than 5 hours with the microscope and computer, respectively. About 67% of pathologists worked according to an organized schedule, and 72% of them were able to manage their daily work on time. General characteristics of the participants are presented in Table 1.

Characteristics related to the workplace, including the features of the microscope, chair, and desk, of the study participants are summarized in Table 3.

**Table 3.** The Characteristics related to the workplace in the study participants

Parameter	Value (N=350)
Workplace atmosphere	
Very good	67 (19.1)
Good	189 (54)
Fair	84 (24)
Poor	10 (2.9)
Workplace features	
Having a window	270 (77.1)
Proper ventilation	200 (57.1)
Shared room	235 (67.1)
Microscope	
Condenser lens	292 (83.4)
Frequently serviced	104 (29.7)
Proper horizontal line of sight	275 (78.6)
Chair	
Adjustable height of the back	245 (70)
Use of ergonomic chair	96 (27.4)
Desk	
Adjustable height	34 (9.7)
Proper size	219 (62.6)
Adjustable inclination and slope	19 (5.4)

## Discussion

The present study showed a high frequency of occupational health problems among the participating pathologists. Musculoskeletal problems were the most prevalent health problems, followed by visual refractive errors, acute injuries, and intolerance reactions to chemicals. 

Musculoskeletal problems as the most prevalent problems in our study were present in 87% of the participants, and 63% of them experienced at least one episode of musculoskeletal pain within the last four weeks. This finding is comparable to the prevalence of various musculoskeletal problems among the general population and other professions, ranging from 25% to 93% (25-28). There was no difference between the genders, but surprisingly, the frequency of musculoskeletal disorders was significantly higher in the younger age groups (*P*=0.004). A higher rate of musculoskeletal problems among the younger participants was against the role of aging for this condition, signifying the impact of occupational factors on the pathologists for developing musculoskeletal problems (29, 30). These findings emphasize the need for conducting preventive measures at a younger age and informing them about working in awkward postures. Lower frequency of symptoms in those who performed an intermittent fasting and stretching exercise during work time and a higher frequency of symptoms in those who worked more with a computer are excellent pieces of evidence for the positive effect of preventive measures. Our findings are in line with those of Fritzsche *et al.* (19), both for the frequency of the musculoskeletal problems and the location of the pain. However, in another study on laboratory professionals in India, the prevalence of symptoms of musculoskeletal disorders was 21.2%, with the neck and the lower back being the most common sites of pain (31). Longer hours of microscope use and the duration of work without breaks were also shown to be associated with musculoskeletal pain, particularly in the neck, in the laboratory workers (9). Similarly, our data showed that those who had fewer working hours, had less microscope use, used ergonomic furniture, and performed regular exercise had less musculoskeletal complaints. 

In a 24-month cohort of 4006 workers in various fields, arm pain was predicted by highly repetitive work while low back pain and lower limb pain were predicted by prolonged standing and the pushing task of heavy objects, respectively (25). Besides postural issues, psychosocial factors of workplace, job satisfaction, and high body mass index could predict the development of musculoskeletal problems during the follow-up period. In our study, neck, lower back, and shoulder pains were the most common musculoskeletal complaints, and as compared with the above-mentioned study, one can judge that they may stem from postural mal-alignments and not using ergonomic furniture. 

Based on the current evidence, visual disturbances (myopia, hyperopia, and other visual refractive errors) among pathologists are more prevalent than that in other health care professionals (32, 33). Our study also showed a high frequency of visual disturbances (~78%), mostly myopia (44% of all affected cases). In another study, the frequency of refractive errors was reported 42%, mostly myopia (observed in 9%) (34). Fifty percent of our participants had visual errors before working as a pathologist, which is much lower than that in previous studies (19). This can be rationalized by the differences in overall frequency of refractive errors in various regions; the prevalence of refractive errors in Europe is more than 50%, much higher than its prevalence in Iran (31, 35, 36). However, one should not forget other factors such as a higher proportion of ametropic medical students who choose pathology as their specialty and long hours of eye-straining activities (i.e., microscopy and computer work) (19, 37). 

On the other hand, 51% of our participants declared that their refractive error was exacerbated by their working period. Although this finding is parallel to that of Fritzsche *et al.* (19), it may reflect the progressive nature of refractive errors through time and by aging process (38, 39). 

Acute injury by sharp instruments during work is an inevitable part of the pathology. In our study, almost 75% of the participants had at least one incidence of acute injury. The cutting injury was the most common type of injury, and the needle stick was the least common type. Residents experienced a significantly higher prevalence of cutting injury by sharp instruments than the specialists had, which is comparable to the findings of Fritzsche *et al.* (19). This finding can be due to the lack of enough experience in the residents and not being familiar enough with the protective measurements. 

Cut-resistant gloves are highly suggested to prevent cut injuries, but they have low compliance and are seldom used (24). Our participants were generally not familiar with cut-resistant gloves, and therefore, we suggested educational programs on protective and preventive devices for pathologists to inform them about the latest advances in this. 

Intolerance reactions to formaldehyde, as a highly used fixator in Pathology Institutes, were reported in 63.3% of our participants, which included severe skin and mucosal irritation, mucosal inflammation, fatigue, and drowsiness. The rate of our study was much higher than that of Fritzsche *et al.* (~25%) and was more observed among the residents. This difference stems from poorly ventilated labs, lower levels of standards during work, and also more spending of time in a low standard workplace by residents.

Due to the high coverage of hepatitis B vaccination in Iran, 96.9% of our participants reported immunization against hepatitis B, and none reported a history of Hepatitis B disease. This rate is higher than the one reported in Switzerland (24) but still demands improvement. 

Common medical conditions in our study population included depression (15.4%), burnout (10.3%), and hypertension (4%) that had similar frequencies in the study of Fritzsche *et al.* (19). However, we did not observe any association between these conditions and the age of the participants. Depression was more frequent among men, while burnout was associated with longer work hours per week. In a similar study, the frequency of depression among pathologists was 6.7%, which is much lower than that in our study (19). This difference should be interpreted under the light of the prevalence of depression among general population where the study sample comes from. However, one should not forget the importance of depression among health care workers and its burden and problems for both the clinicians and the patients. In addition, depression was less in participants who worked in university hospitals; this could be explained by the interactive relationship between the residents and specialists and the academic environment of the workplace. Hypertension was more prevalent among older participants. Only five participants had malignancy, but it did not seem to be associated with formalin exposure. One pathologist had tuberculosis that is much lower than that in previous reports (24, 40, 41). However, we recommend tuberculosis-screening strategies as a cost-effective measure for pathologists, as well as all health care workers (42). 

Hepatitis B virus (HBV) vaccination had a high coverage in our study population, and interestingly, it was higher than the rate reported for the pathologists in Switzerland (19). This finding shows that previous immunization programs in Iran, either for general population or health care professionals, were successfully performed. However, due to the importance of immunization among health care professionals, we recommend screening programs to increase the vaccination coverage to 100% in this high-risk group. 

Finally, a majority of our participants had a bright perspective on working as a pathologist and forecasted a positive work future for the next five years. Therefore, we believe investment in improving the personal health of the pathologists is crucial, and it can enhance their occupational output and personal health.

Our study has several strengths. First, it was the first study on the occupational health of pathologists in Iran and the Middle East. Second, it included the largest English study sample as compared with previously published studies in the world, hence our data can effectively contribute to a systematic review or meta-analysis in the future. 

Nonetheless, we had some limitations, as well. This was a cross-sectional study, and therefore it only encompassed data at a certain time. Therefore, one cannot describe the changes in the health of pathologists through time or find cause and effect relationships between occupational factors and the health of the participants. Second, most of our participants were from the capital city, and a broader study with more participants from smaller cities could give a better picture of the occupational health of the pathologists in Iran. 

## Conclusion

As the first study on the occupational health of pathologists in Iran, we observed a noticeable frequency of health risks and complications among our participants. These problems included musculoskeletal problems, visual errors, acute injuries, chemical exposure, and medical conditions such as depression. Due to the higher frequency of some of these problems among the residents, we emphasize the need for promoting protective and preventive measures from the beginning of their career as a pathologist. We believe that establishing a thorough occupational health care program for the pathologists as highly trained medical professionals, as well as other medical professionals, and implementing preventive and protective measures could help to improve their health.
